# Evidence for a novel Kit adhesion domain mediating human mast cell adhesion to structural airway cells

**DOI:** 10.1186/s12931-015-0245-z

**Published:** 2015-07-15

**Authors:** Kevin C. Gough, Ben C. Maddison, Aarti Shikotra, Elena P. Moiseeva, Weidong Yang, Shila Jarvis, Peter Bradding

**Affiliations:** School of Veterinary Medicine and Science, The University of Nottingham, Sutton Bonington Campus, College Road, Sutton Bonington, Leicestershire, LE12 5RD UK; ADAS UK, School of Veterinary Medicine and Science, The University of Nottingham, Sutton Bonington Campus, College Road, Sutton Bonington, Leicestershire, LE12 5RD UK; Department of Infection, Immunity and Inflammation, Institute for Lung Health, University of Leicester, Leicester, UK; ADAS UK, Biology Department, University of Leicester, University Road, Leicester, LE1 7RH UK; Department of Respiratory Medicine, Glenfield Hospital, Groby Rd, Leicester, LE3 9QP UK

**Keywords:** Mast cell, Airway epithelium, Airway smooth muscle, Phage display, ScFv, Kit (CD117)

## Abstract

**Background:**

Human lung mast cells (HLMCs) infiltrate the airway epithelium and airway smooth muscle (ASM) in asthmatic airways. The mechanism of HLMC adhesion to both cell types is only partly defined, and adhesion is not inhibited by function-blocking anti-Kit and anti-stem cell factor (SCF) antibodies. Our aim was to identify adhesion molecules expressed by human mast cells that mediate adhesion to human ASM cells (HASMCs) and human airway epithelial cells.

**Methods:**

We used phage-display to isolate single chain Fv (scFv) antibodies with adhesion-blocking properties from rabbits immunised with HLMC and HMC-1 membrane proteins.

**Results:**

Post-immune rabbit serum labelled HLMCs in flow cytometry and inhibited their adhesion to human BEAS-2B epithelial cells. Mast cell-specific scFvs were identified which labelled mast cells but not Jurkat cells by flow cytometry. Of these, one scFv (A1) consistently inhibited mast cell adhesion to HASMCs and BEAS-2B epithelial cells by about 30 %. A1 immunoprecipitated Kit (CD117) from HMC-1 lysates and bound to a human Kit-expressing mouse mast cell line, but did not interfere with SCF-dependent Kit signalling.

**Conclusion:**

Kit contributes to human mast cell adhesion to human airway epithelial cells and HASMCs, but may utilise a previously unidentified adhesion domain that lies outside the SCF binding site. Targeting this adhesion pathway might offer a novel approach for the inhibition of mast cell interactions with structural airway cells, without detrimental effects on Kit signalling in other tissues.

## Background

Mast cells (MC) play a significant role in the pathophysiology of many diverse diseases including asthma [1]. Interestingly in asthma, MCs infiltrate the airway smooth muscle (ASM) [2], bronchial epithelium [3] and submucosal glands [4], and within these sites demonstrate features of activation. MC mediator release therefore occurs within these dysfunctional airway elements and has a profound effect on airway function, inflammation and remodelling [1]. Targeting the interactions between MCs and structural airway cells therefore has the potential to offer a novel approach to the treatment of asthma.

Adhesion is a fundamental mechanism through which cells communicate allowing the targeting of specific cell-to-cell signals. Human lung mast cells (HLMC) and the immature leukaemic human MC line HMC-1 adhere avidly to both primary human ASM cells (HASMCs) [5] and primary human airway epithelial cells [6], and both MC types display identical adhesion properties [5, 6]. The mechanisms of adhesion however remain poorly defined. With respect to the bronchial epithelium, the adhesion mechanism is unknown but does not utilise recognised integrins and C-type lectins [6]. Similarly, constitutive HLMC and HMC-1 adhesion to HASMCs does not involve recognised integrins or their ligands [5]. Unlike adhesion to airway epithelium, HLMC adhesion to HASMCs is partially dependent on Ca^2+^, and thus resembles adhesion of skin MCs and HMC-1s to skin fibroblasts [5, 7]. Of note, human MC-epithelial and MC-HASMC adhesion were not inhibited by function blocking anti-Kit (stem cell factor [SCF] receptor, CD117) or anti-SCF antibodies [5, 6]. However, human HASMC-MC adhesion is mediated in part via cell adhesion molecule-1 (CADM1) [5], and when HLMCs and HASMCs are activated with TGFβ and TNFα respectively, there is enhancement of HLMC adhesion which is mediated via CD51/61 [8].

The importance of these adhesive interactions is demonstrated by observations that HASMCs maintain HLMC survival and induce their proliferation through a CADM1-dependent mechanism [9]. Inhibiting adhesion therefore has great potential for the attenuation of a wide range of allergic and fibrotic disorders. To further define the mechanisms mediating HLMC adhesion to HASMCs and bronchial epithelial cells, we have used the technique of phage-display to express a library of recombinant antibodies raised against human MC plasma membranes. Using this technique we have isolated a single chain Fv (scFv) antibody that binds to Kit but does not modulate its signalling, and which partially inhibits HMC-1 adhesion to both HASMCs and bronchial epithelium.

## Methods

### Ethics statement

All patients donating tissue gave written informed consent. Use of tissue from lung resection was approved by the National Research Ethics Service (reference 07/MRE08/42) and bronchoscopic studies were approved by the Leicestershire Research Ethics Committee (reference number 4977).

### Human mast cell purification and culture

HLMCs were isolated and cultured as described previously [6, 10]. Final purity was >99 %, and viability >97 % in all experiments.

The leukaemic human MC line HMC-1 (Dr J Butterfield, Mayo Clinic, Rochester, USA) was cultured as described previously [6].

### Human airway smooth muscle cell and BEAS-2B culture

HASMCs were isolated using explant culture of ASM bundles as previously described [11]. HASMCs were cultured in DMEM supplemented with 10 % FBS, antibiotic/antimycotic agents and non-essential amino acids [11]. BEAS-2B epithelial cells were purchased from the European Collection of Animal Cell Cultures (Porton Down, Wiltshire, UK). Cells (passages 8–12) were grown on human plasma fibronectin-coated T75 culture flasks in BEBM media (Clonetics Cat. No. CC4175), with an added enhancement bullet kit (Clonetics Cat. No. CC4175), Pen/Strep (5 ml) and fungizone (5 ml) to create basal epithelial growth media (BEGM). BEAS-2B were then passaged on to human plasma fibronectin-coated 96-well plates and then grown to confluence prior to use in adhesion assays as described previously [6].

### Micro-organisms and plasmids

Escherichia coli XL1-blue (endA1 gyrA96 (nal^R^) thi-1 recA1 relA1 lac glnV44 F’[::Tn10 proAB^+^ lacI^q^ Δ (lacZ) M15] hsdR17 (r_K_^−^ m_K_^+^)) and HB2151 (K12, ara, del (lac-pro), thi/F’proA + B+, lacIq, lacZdelM15) were obtained from Pharmacia LKB (St Albans, Herts, UK). Gene Hogs competent cells (F- *mcr*A Δ (*mrr-hsd*RMS-*mcr*BC) φ80*lac*ZΔM15 Δ*lac*X74 *rec*A1 *ara*D139 Δ (*ara-leu*) 7697 *gal*U *gal*K *rps*L (StrR) *end*A1 *nup*G *fhu*A::IS2 (confers phage T1 resistance) were obtained from Invitrogen (Paisley, UK). Ex-12 helper phage [12] was obtained from Ig Therapy Co. (College of Agriculture of Life Sciences, Kangwon National University, Republic of Korea). The construction of the phagemid vector pSD3 is described by Li et al. [13].

### Preparation of HLMC and HMC-1 membranes

HLMCs from several donors were suspended in buffer (10 mM Tris–HCl, pH 7.4, 1 mM EDTA, 200 mM sucrose, 1 mM phenylmethylsulfonyl fluoride) and homogenized. The nuclei and cell debris were removed from the homogenate by centrifugation (900 g, 10 min, 4 °C). The pellet was washed with buffer and centrifuged (900 g, 10 min). The resulting supernatants were pooled and centrifuged (110000 g, 75 min, 4 °C). The supernatant was discarded, the membrane pellet dissolved in PBS, and protein concentration was measured (Bio-Rad DC protein assay, BioRad, Hemel Hempstead, UK). 1.54 mg of HLMC membrane protein was obtained from 6.5 × 10^7^ cells. Using the same protocol, 4.6 mg of membrane protein was obtained from 1 × 10^8^ HMC-1 cells.

### Immunisation of rabbits with mast cell membranes

Two female New Zealand white rabbits were immunised with a 1 ml mixture of 0.4 mg HLMC membrane protein in 0.5 ml PBS and 0.5 ml Titermax Gold. Three weeks later, both rabbits were boosted with a mixture of 0.25 mg HLMC membrane protein and 0.5 mg HMC-1 membrane protein. After a further two weeks, both rabbits were boosted with 0.7 mg HMC-1 membrane protein. After a further two weeks, both rabbits were boosted with 0.12 mg HLMC membrane protein and 0.5 mg HMC-1 membrane protein. Post-immune sera were labelled R91 and R92.

Experiments using rabbits were carried out within the Biomedical Services Unit at the University of Leicester, UK. All animal procedures were performed under Home Office (UK) and local ethical review committee approval and compliant with the Animal (Scientific Procedures) Act 1986. The rabbits were housed in scanabur rabbit cages and fed on SDS Stanrab diet. They were on a light cycle of 12 h light, 12 h dark (7 am-7 pm). Local anaesthetic cream was applied prior to blood sampling. Terminal procedures: overdose of anaesthetic with exsanguination to confirm death.

### Generation of an immune rabbit scFv-phage library

Protocols for DNA manipulation were taken from Sambrook et al. [14] or were those recommended by the manufacturers. The construction of an immune phage-display single chain variable region (scFv) antibody library was carried out as described by Gough et al. [15] and Kuhne et al. [16]. Briefly, the spleens from both rabbits were cut into thin pieces, submerged in RNAlater and stored at 4 °C. Total RNA was isolated from spleen tissue using a RNA isolation kit (Qiagen, Surrey, UK) and used in the production of first strand cDNA using a cDNA synthesis kit (Pharmacia, Herts, UK) and a random hexa-nucleotide primer. The VL and VH repertoire of the rabbits were amplified by PCR. Purified VL PCR products were cleaved with SfiI and purified VH PCR products cleaved with PflMI. Cleaved products were purified by gel extraction and the VL repertoire ligated into SfiI cut pSD3 [13]. VL-pSD3 ligation products were dialysed against sterile water and then 42 ng used to transform GenHogs OneShot competent cells. Multiple transformations were carried out yielding 3 × 10^6^ VL-pSD3 containing cells. VL fragment diversity was assessed by amplification of the VL gene using primers pSDF (5’-TATTTCAAGGAGACAGTC-3’) and pSDseq2 (5’-AACCCACTCCTTGGCCTTC-3’) followed by digestion of the PCR products with BstNI restriction enzyme. Resulting fragments were analysed on a 3 % (w/v) agarose gel. The ‘VL library’ had an estimated diversity of 2.3 × 10^6^ distinct light chains. The VL-pSD3 library was cleaved with PflMI and the VH repertoires ligated in. Purified pSD3:scFv ligation product (40 ng) was then used to transform electrocompetent GenHogs OneShot cells. ScFv fragment diversity was assessed by amplification of the scFv gene using primers pSDF and pSDR (5’-ATTGGCCTTGATATTCAC-3’) followed by analysis of DNA fragments produced upon BstNI digestion. Multiple transformations yielded a scFv library consisting of an estimated 3.7 × 10^7^ different antibodies. The library DNA was purified and transformed into XL1-blue host cells. The resulting clones coded for scFv-pIII fusion proteins containing poly-Histidine and C-myc tags between the scFv and pIII domains. An amber top codon was situated between the C-myc tag and the pIII gene [13].

### Selection of mast cell-specific scFv-displaying phage

Phage were prepared from the rabbit anti-mast cell library as described by Oh et al. [12] utilising Ex-12 helper phage to rescue scFv-displaying phage. Phage selection was carried out in solution; HMC-1 cells were pelleted at 500 g and washed twice in RPMI (Sigma, Poole, UK), re-pelleted and then resuspended in 1 ml Hank’s buffered salt solution (HBSS, 0.137 M NaCl, 5.4 mM KCl, 0.25 mM Na_2_HPO_4_, 0.44 mM KH_2_PO_4_, 1.3 mM CaCl_2_, 1.0 mM, MgSO_4_, 4.2 mM NaHCO_3_). The phage library was then depleted of antibodies that display binding to antigens on the cell surface that are not recognised by the immune sera; this was carried out by binding the phage scFvs to HMC-1 cells that had been epitope-masked as described by Popkov et al. [17]. Briefly, 0.5 ml of the HMC-1 cells (1-2 × 10^7^ cells) were incubated with 200 μl of a pool of the anti-mast cell serum R91 and R92 for 30 min at room temperature before cells were blocked with 2 % (w/v) Marvel-HBSS for 30 min at RT. Cells were then washed 3 times in HBSS and resuspended in 1 ml of blocked phage solution. For the initial round of panning, PEG-precipitated library phage (5 × 10^13^/ml, 1 ml) were blocked in 3 % (w/v) Marvel-HBSS for 45 min at RT. For subsequent rounds, 1 ml of the supernatant of an overnight bacterial culture of phage was used. Blocked phage were incubated with the epitope-masked HMC-1 cells for 30 min at room temperature with rotating. Cells were pelleted (500 g, 5 min) and the phage-containing supernatant removed (‘depleted phage’). ‘Unmasked’ HMC-1 cells (1-2 × 10^7^ cells) were blocked in HBSS-3 % (w/v) Marvel for 30 min at RT. ‘Unmasked’ cells were then pelleted and resuspended in the ‘depleted phage’ and incubated for 1 h at RT with rotating. Unbound phage were removed by washing 3 times with RPMI and 3 times with HBSS; bound phage were then eluted in 25 mM triethylamine as described previously [15]. Up to four rounds of iterative scFv-phage selection were carried out with each round including a negative selection of phage against epitope-masked HMC-1 cells followed by a positive selection against unmasked HMC-1. Monoclonal phage-displayed scFvs were obtained after 3 or 4 rounds of panning by plating out the XL1-blue infected cells to single colonies. Cultures of these single colonies were then used to produce monoclonal scFv-phage particles by rescue with Ex-12 helper phage. For the production of soluble scFvs, monoclonal scFv-phage particles were used to infect HB2151 host cells followed by IPTG induced expression [15].

### Phage-scFv ELISA

Both polyclonal and monoclonal phage preparations were assayed against HMC-1 cells by ELISA. Polyclonal phage were assayed at 1 × 10^11^/ml and monoclonal phage were assayed using 100 μl of supernatant from an overnight culture of bacteria following phage rescue. All steps were carried out within deep well tissue culture plates and the final step transferred to Nunc Maxisorb ELISA plates (Gibco BRL, Nunc products) to allow absorbance readings to be taken. All blocking and wash steps were as in the panning protocol. Phage-scFv were bound to HMC-1 cells (~10^6^ cells per well) for 1 h at room temperature, bound phage were detected in two steps using biotin labelled anti-fd antibody (1/160) and then extravadin-AP conjugate (1/10,000; both from Sigma). Signals were developed with PNPP substrate from Sigma and the absorbance at 405 nm measured.

### ScFv purification

Soluble scFv was purified via its poly-His tag on a HisPur Cobalt resin column as recommended by the manufacturers (Pierce). ScFv concentration was estimated using the BCA protein assay kit (Pierce, Rockford, IL, USA) and protein purity assessed by analysis by sodium dodecyl sulphate-polyacrylamide gel electrophoresis (SDS-PAGE). Pure scFvs were stored in 37.5 % (v/v) glycerol at −80 °C at concentrations in the range 0.1 to 0.9 mg/ml.

### SDS-PAGE and Western blotting

Discontinuous SDS-PAGE was carried out through a running gel containing 12 % (w/v) total acrylamide using NuPAGE pre-cast Bis-Tris gels and Seeblue Plus 2 protein markers (Invitrogen, Paisley, UK). Electrophoresed gels were stained using 0.05 % (w/v) Coomassie Brilliant Blue R250 (Fischer Scientific). For Western blots, separated proteins were transferred to polyvinylidine difluoride membrane (Roche) using a NuPAGE Blot module (Invitrogen) at 30 V for 1 h. The 9E10 anti-C-myc antibody was used to identify scFvs. For staining of Kit in Western blots, a mouse IgG1 anti human Kit antibody (E1, Santa Cruz Biotechnology Inc., Santa Cruz, CA, USA) was used.

### Identification of scFv amino acid sequences

ScFv clones were sequenced by dye termination with AmpliTaq DNA polymerase, FS on a 377 ABI automated DNA sequencer using the primers pSDF (5’-TATTTCAAGGAGACAGTC-3’) and pSDR (5’-ATTGGCCTTGATATTCAC-3’). The scFv sequences were aligned as described by Kabat *et al.* [18]. The antibodies consisted of a VH-a1 heavy chain [19] combined with a kappa light chain.

### Flow cytometry

MCBS1 mouse mast cells were a kind gift from Dr Dean Metcalfe, National Institute for Allergy and Infectious Diseases, NIH, Bethesda, MD) [20]. Control non transfected cells, mock transfected cells (E1-AA685) or human Kit-transfected cells (W1-AA677) were stained with 4 ug/mL PE-labelled anti-Kit mAb (BD Bioscience, Oxford, UK) or 5 μg/mL A1 scFv antibody followed by 9E10 (anti-myc) secondary antibody, which was then indirectly labelled with R-Phycoerythrin (PE)-labelled rabbit anti-mouse antibody (Dako, UK). Appropriate isotype controls were performed (mouse mAb IgG1-PE (BD Bioscience, Oxford, UK) or E4 scFv isotype). Staining was analysed by one colour flow cytometry on a FACSCanto (BD Biosciences, Oxford, U.K.). The same protocol was used for analysis of scFv binding to HMC-1 cells and HLMCs where bound scFv was detected with anti-C-myc 9E10, and then labelled with FITC-labelled rabbit anti-mouse antibody (Dako, Ely, UK), or RPE-labeled rabbit anti-mouse (Dako) as described previously [21]. HMC-1 cells were pre-incubated with SCF 100 ng/ml for 15 min to assess the effect of Kit internalisation on scFv binding.

To detect polyclonal sera binding to HLMCs, the same protocol was performed but using 10^5^ mast cells and 10 μl of 1:10 to 1:10,000 dilutions of polyclonal sera, and using PBS-0.1 % (w/v) BSA buffer throughout. Bound polyclonal antibody was detected with anti-rabbit IgG-FITC (1:10 dilution).

### Immunofluorescent staining

W1-AA677, E1-AA685 and control MCBS1 mouse mast cells were grown on fibronectin-coated chamber slides and labeled with the appropriate mAb or isotype control as used for flow cytometry. A1 antibody was indirectly labeled with 9E10 anti-myc secondary mouse mAb and then RPE-labeled rabbit anti-mouse (Dako). Cells were counterstained with 4′,6′-diamidino-2 phenylindole (DAPI, Sigma, Gillingham, Dorset, UK) and the slide was mounted using fluorescent mounting medium. Cells were visualized using a computer imaging system (Cell F, Olympus, Germany).

### Adhesion assays

Based on saturation of staining identified using flow cytometry, polyclonal pre- and post-immune rabbit sera were incubated with HLMC cells at a 1:10 dilution, and scFvs with HMC-1 and HLMCs at approximately 20 μg/ml for 30 min at room temperature. HLMCs and HMC-1 cell adhesion to BEAS-2B epithelial and primary HASMCs was then assessed as described previously [5, 6].

### Immunoprecipitation of scFv-bound mast cell ligand

For immunoprecipitation experiments, anti-C-myc 9E10 was covalently coupled to protein A/G Agarose using the Pierce Crosslink Immunoprecipitation kit (Pierce) using the manufacturer’s instructions. ScFv A1 and E4 (80 μg) were then bound to 80 μl of 50 % (v/v) 9E10-proteinA/G agarose resin in 0.01 M sodium phosphate, 0.15 M NaCl; pH 7.2 for 16 h at 4 °C. Resin was washed 3 times in PBS and twice in lysis/wash buffer. HMC-1 membrane pellets were prepared as described above from 1.6 × 10^7^ cells and then solubilised in 1.2 ml of lysis/wash buffer (0.025 M Tris, 0.15 M NaCl, 0.001 M EDTA, 1 % NP-40, 5 % glycerol, pH 7.4) by incubation on ice for 20 min. Samples were centrifuged (17000 g, 20 min, 4 °C) and supernatants collected. Pellets were resuspended in the same buffer and incubated and centrifuged as before. Supernatant was collected and pooled with the previously obtained supernatant. Soluble native HMC-1 membrane (400 μl) was applied to the scFv-9E10-protein A/G agarose resin and allowed to bind at RT for 5 h with rotating. In spin columns, the resin was centrifuged (800 g, 10 s), resin was then washed 4 times with 500 μl TBS and once with 200 μl of conditioning buffer (Pierce Crosslink Immunoprecipitation kit). Protein was then eluted in three 100 μl volumes of a low pH elution buffer (Pierce Crosslink Immunoprecipitation kit). Immunoprecipitated proteins were separated on SDS-PAGE gels and visualised by staining with Coommassie brilliant blue. The A1 specific band of interest was excised and analysed by in-gel trypsin digestion followed by peptide mass fingerprint using MALDI-ToF mass spectrometry (service run by the Protein Nucleic Acid Chemistry Laboratory, University of Leicester, UK).

### Assessment of Kit phosphorylation in HMC-1

10^6^ HMC-1 cells in 1 ml were treated with 20 μg/ml E4 or A1 antibody for 15 min at 37 °C, followed by 100 ng/ml SCF for 3 min at 37 °C; 30 mg/lane of protein extract was resolved in 4–12 % gradient SDS-PAGE gel transferred on 2 blots, probed with either pY99 [Santa Cruz, sc-7020], anti-Kit E1 [Santa Cruz, sc-17806] and beta-actin C4 [Santa Cruz, sc-47778].

### Statistical analysis

Differences in adhesion between experimental conditions were examined using paired t tests where appropriate. *p* < 0.05 was considered statistically significant.

## Results

### Polyclonal sera binding to HLMC and inhibition of HLMC adherence to BEAS-2B bronchial epithelial cells

**Fig. 1:**
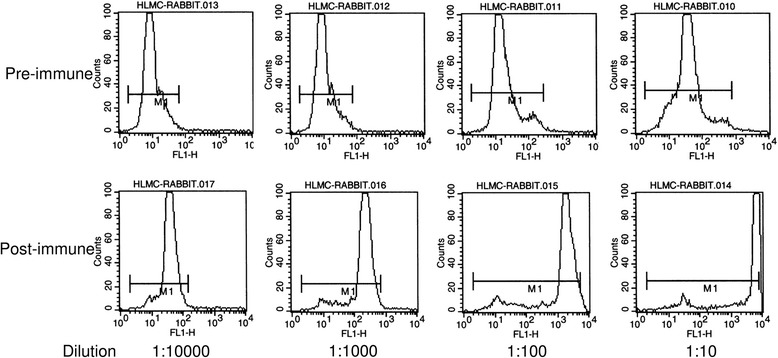
Binding of post immune rabbit sera to HLMC. Flow cytometry showing that the post immune serum from a rabbit immunised with HLMC/HMC-1 plasma membranes (bottom row) demonstrates increased binding to HLMC compared to the pre-immune serum (top row) at equivalent dilutions. Representative of data from two immunised rabbits

**Fig. 2:**
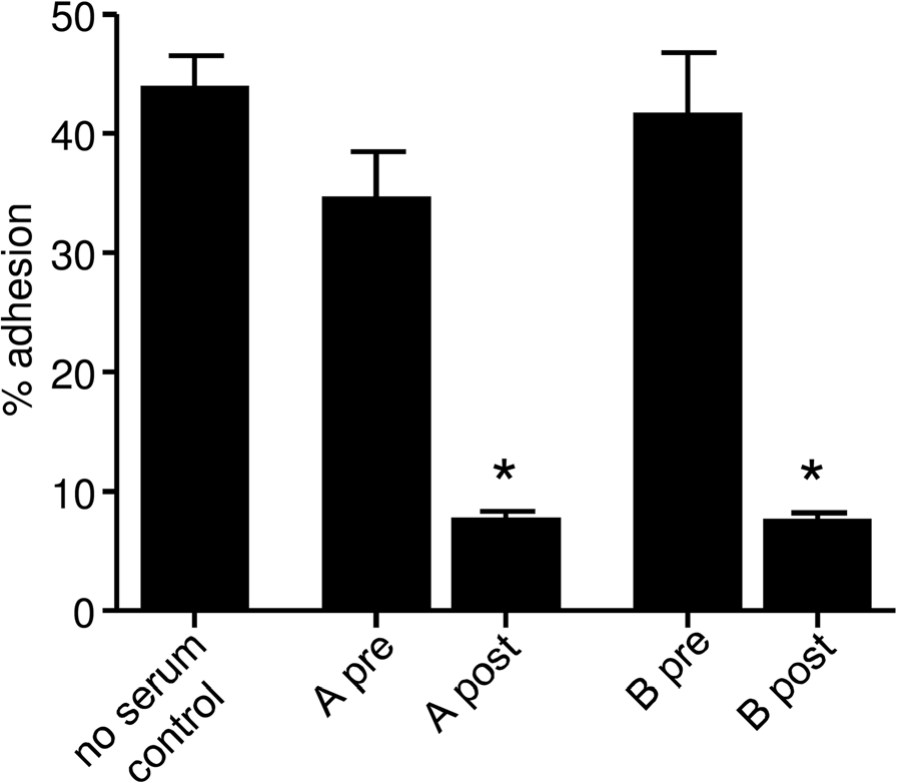
Inhibition of HLMC adhesion to BEAS-2B by post immune rabbit sera. Adhesion of HLMC to BEAS-2B human bronchial epithelial cells was attenuated by the post immune serum from two rabbits (A and B) immunised with HLMC/HMC-1 plasma membranes. *n* = 5 HLMC donors studied in 5 independent experiments. *p = 0.004 for each

The polyclonal serum collected from two post-immune rabbits labelled HLMCs clearly at dilutions from 1:10 to 1:10,000 when compared to control pre-immune serum (Fig. 1). In addition, the post-immune sera from both rabbits markedly inhibited HLMC adhesion to BEAS-2B epithelial cells compared to pre-immune sera at a 1:10 dilution (*n* = 5 HLMC donors, *p* = 0.004 for both rabbits; Fig. 2). The post-immune sera did not enhance histamine release from HLMCs compared to the pre-immune sera (data not shown).

### ScFv-phage library production and selection of anti-human mast cell scFv antibodies

**Fig. 3:**
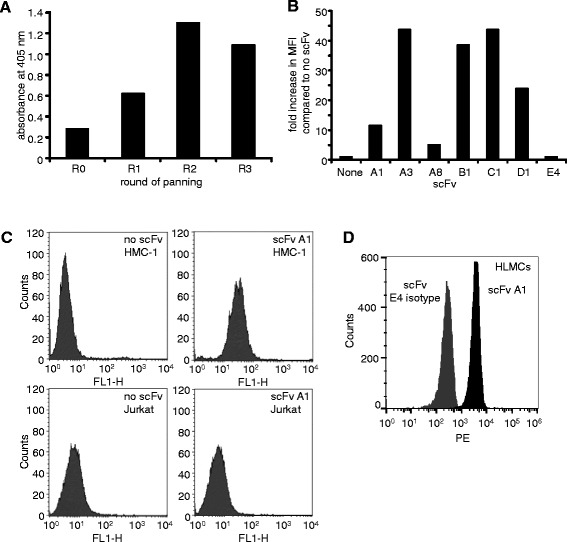
The isolation of scFvs that bind to mast cells. **a** Binding of polyclonal phage-displayed antibodies to HMC-1. Three rounds of panning were carried out, with each round including a negative selection of phage against epitope-masked HMC-1 cells, followed by a positive selection against unmasked HMC-1. HMC-1 cells were detected with 10^10^ phage from panning rounds 0, 1, 2, and 3 (R0 to R3). Bound scFv was detected with biotin labelled anti-fd antibody and then extravadin-AP conjugate. Absorbances were measured at 405 nm. Individual scFvs that bound to HMC-1 cells were selected by phage ELISA. **b** and **c** scFv binding was confirmed by flow cytometry. HMC-1 or Jurkat cells were incubated with purified scFv and binding was visualised with anti-C-myc 9E10 antibody and anti-mouse antibody-FITC conjugate. The mean fluorescence produced with each scFv is expressed as a fold-increase from that produced in the absence of scFv **b**. ScFvs A1, A3, A8, B1, C1 and D1 were selected against HMC-1; the scFv E4 is a control scFv which does not bind to HMC-1. Representative examples of flow cytometry histograms for the binding of scFvs to HMC-1 and Jurkat cells are shown for antibody A1 and for a no scFv control **c**. The binding of scFvs to HMC-1 was repeated a minimum of 4 times and all 6 scFvs demonstrated clear binding to HMC-1 cells on each occasion. **d** scFv A1 also bound to HLMCs when compared to E4 (representative of 3 independent experiments using 3 HLMC donors)

An scFv library estimated to contain approximately 3.7 × 10^7^ different antibodies was produced from spleen cells of the two rabbits immunised with HLMC and HMC-1 membrane fractions. ScFv-phage antibodies were selected from the library over 3 or 4 rounds of selection in two independent experiments. The polyclonal scFv-phage isolated after each round of selection clearly demonstrated enrichment for binders to the HMC-1 cells (Fig. 3a and data not shown). Individual phage clones were isolated from panning rounds with a clear enrichment for binders to HMC-1 (rounds 3 and 4). Monoclonal scFv were assessed by phage-ELISA and those producing ELISA signals at least twice the background signal (produced in the absence of scFv-phage) were selected and the scFv genes sequenced. Unique scFvs from these candidate mast cell binders were produced as soluble antibodies within HB2151 host cells and purified antibodies were assessed for binding to HMC-1 by ELISA and flow cytometry. Six distinct scFvs were identified that bound to HMC-1 and did not bind to control Jurkat cells (Fig. 3b and c and data not shown).

### scFv A1 inhibits HMC-1 adhesion to HASMCs and BEAS-2B epithelial cells

**Fig. 4:**
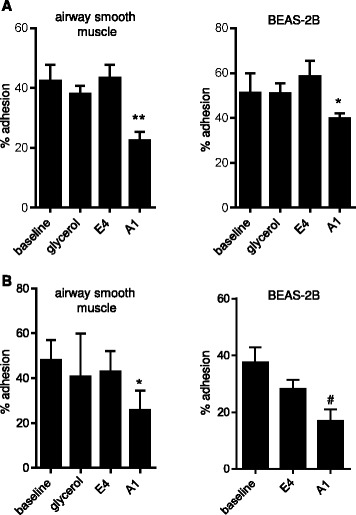
The effects of scFv A1 on mast cell adhesion. **a** HMC-1 adhesion to monolayers of BEAS-2B human airway epithelial cells and primary HASMCs was assessed in the presence of anti-mast cell scFv A1 or a control scFv E4 which does not bind. Baseline adhesion and adhesion in control media containing antibody vehicle (glycerol, final concentration 0.8-1.5 % depending on the scFv batch) is also shown. Plotted as mean ± SEM for all experiments; *n* = 8 ASM donors used in independent experiments and *n* = 4 independent experiments for BEAS-2B. *p = 0.048 compared to E4, **p = 0.008 compared to E4. **b** HLMC adhesion to monolayers of BEAS-2B human airway epithelial cells and primary HASMCs was assessed in the presence of anti-mast cell scFv A1 or a control scFv E4 which does not bind. Baseline adhesion and adhesion in control media containing antibody vehicle (glycerol, final concentration 0.8-1.5 % depending on the scFv batch) is also shown for HASMCs, baseline adhesion is shown for BEAS-2B cells (glycerol not assessed). Plotted as mean ± SEM for all experiments; *n* = 1 ASM donor and 7 HLMC donors used in *n* = 7 independent experiments, and *n* = 6 HLMC donors in independent experiments for BEAS-2B. *p = 0.029 compared to E4, # *p* = 0.017 compared to E4

The final glycerol concentration in the adhesion assays was between 0.8 and 1.5 % depending on the stock concentration of purified antibody. Glycerol did not affect HMC-1 adhesion to either HASMCs or BEAS-2B cells, or HLMC adhesion to HASMCs (glycerol not tested for BEAS-2B cells) (Fig. 4). The six scFvs that displayed binding to mast cells were assessed for their ability to inhibit HMC-1 adhesion to these cell types; the concentrations used were based on saturation of staining identified using flow cytometry. Of these antibodies, purified scFv A1 at a concentration of approximately 20 μg/ml consistently inhibited adhesion of HMC-1 cells to both HASMCs (*n* = 8 donors used in independent experiments) and the BEAS-2B epithelial cell line (*n* = 4 independent experiments) (Fig. 4a). Adhesion of HMC-1 to HASMCs was reduced from 43.4 ± 4.4 % in the presence of a non-binding isotype control scFv (E4) to 22.3 ± 2.9 % in the presence of A1 (*p* = 0.008). Adhesion of HMC-1 to BEAS-2B cells was reduced from 58.4 ± 7.4 % in the presence of E4 control scFv to 39.7 ± 2.4 % in the presence of A1 (*p* = 0.048).

scFv A1 also bound to HLMCs (Fig. 3d). Adhesion of HLMCs to HASMCs was reduced from 42.9 ± 9.2 % in the presence of E4 isotype control, to 25.8 ± 8.7 % in the presence of A1 (*p* = 0.029, 7 independent experiments) (Fig. 4b). Adhesion of HLMCs to BEAS-2B cells was reduced from 28.3 ± 3.1 % in the presence of E4 to 16.9 ± 4.0 % in the presence of A1 (*p* = 0.017, 6 independent experiments) (Fig. 4b). A1 at a concentration of 10 μg/ml was less effective at inhibiting HMC-1 adhesion to HASMCs than 20 μg/ml (30 ± 10 % inhibition [*p* = 0.12], versus 65.3 ± 7.2 % respectively [*p* = 0.001], *n* = 4 independent experiments). Four independent batches of antibodies were used in the HASMC adhesion assays and three independent batches in the BEAS-2B adhesion assays; the efficacy of A1 was similar across batches. Neither A1 nor E4 released histamine from HMC-1 cells or HLMCs (not shown). A1 did not alter HMC-1 viability or cell numbers over 1, 6, 24 or 48 h compared to E4.

### ScFv amino acid sequence

**Fig. 5:**
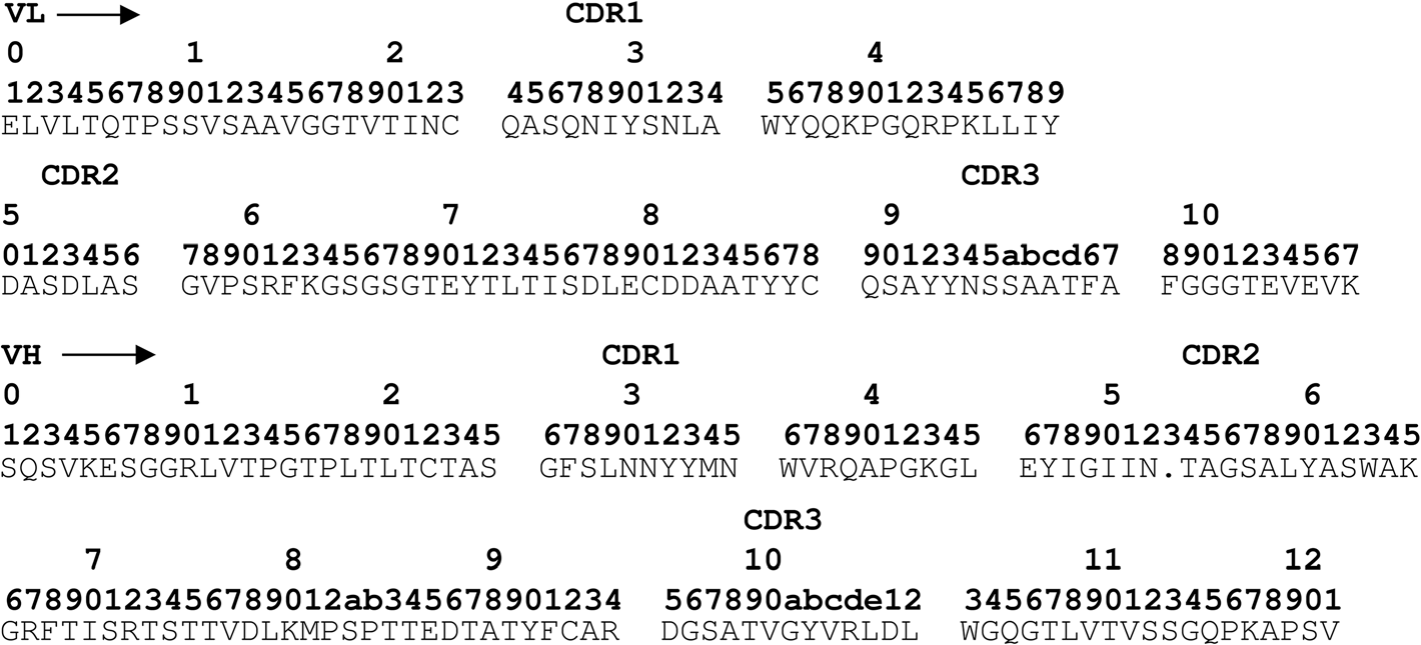
Deduced amino acid sequence of the anti-mast cell scFv A1. The alignment is according to the method of Kabat *et* al. [18]

The anti-mast cell antibody A1 was analyzed by DNA sequencing. The deduced amino acid sequences (Fig. 5) shows that the antibody contains a kappa light chain combined with VH1-a1 heavy chain. The E4 control antibody was also sequenced and also contained a kappa light chain combined with VH1-a1 heavy chain but with distinct CDR regions compared to A1 (data not shown).

### Immunoprecipitation and identification of the scFv A1 cell surface antigen

**Fig. 6:**
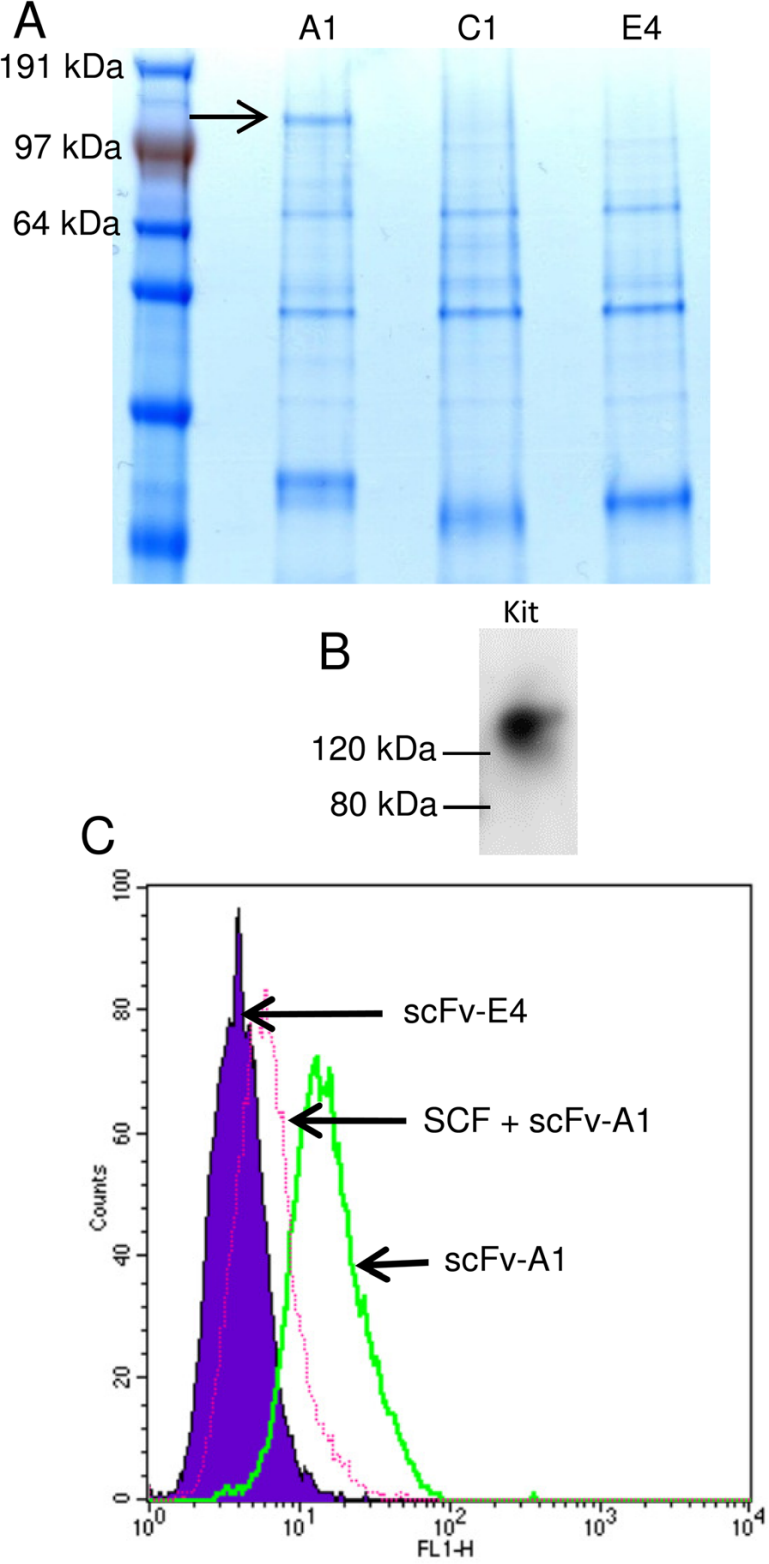
Identification of Kit as the antigen for scFv A1. **a** Immunoprecipitation of HMC-1 plasma membrane proteins with scFv A1 identifed a unique band of approximately 145 kDa (arrow). Analogous immunoprecipitations using scFvs C1 (anti-mast cell scFv) and E4 (scFv with irrelevant binding) are shown for comparison. Excision and sequencing demonstrated that this was Kit. **b** Western blotting of protein immunoprecipitated by scFv A1 demonstrated positive staining using an anti-Kit antibody, with a doublet of the expected size for Kit (145/120 kDa). **c** Flow cytometry histograms demonstrating that following incubation of HMC-1 with SCF (100 ng/ml) for 15 min, binding of scFv A1 was markedly reduced compared to control, in keeping with SCF-induced Kit internalisation

**Fig. 7:**
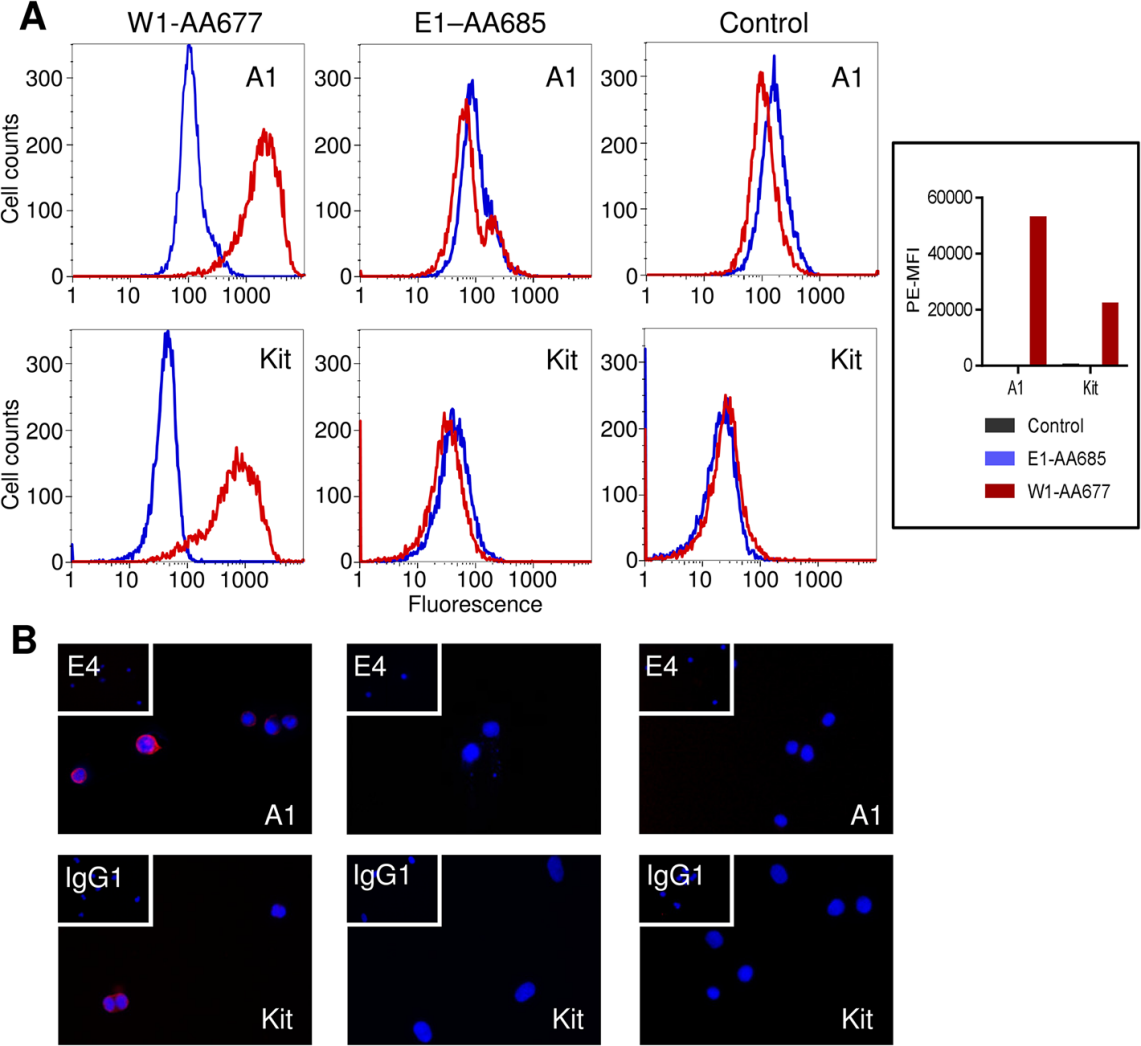
scFv1 A1 binds to human Kit-expressing MCBS1 mouse mast cells. Kit expression was confirmed by flow cytometry (**a**) and immunofluorescence (**b**). **a** Fluorescent histograms (red lines), plotted with corresponding isotype controls (blue line) demonstrate A1 and Kit expression in the W1-AA677 human Kit-expressing mouse mast cell line, but not in E1-AA685 mock-transfected cells or non-transfected control cells. The mean fluorescent intensity was determined minus the total binding of matched isotype controls for A1 and Kit, scFv E4 and mouse IgG respectively. Representative of 3 independent experiments. **b** Immunofluorescent staining of W1-AA677, E1-AA685 and control cells. Nuclei stained blue with DAPI, A1 and Kit stained red (RPE); inset: isotype control. Representative of two independent experiments

To determine what scFv A1 binds to we first examined Western blots of HMC-1 cells probed with A1 but no staining was evident (not shown). We then used A1 for the immunoprecipitation of HMC-1 proteins and obtained a discreet band of approximately 145 kDa which was specific for immunoprecipitation with this scFv (Fig. 6a). Excision and sequencing of this band demonstrated that it was Kit (CD117, SCF receptor). To confirm further that the immunoprecipitated protein was Kit, we demonstrated that a commercial Kit antibody detected the protein precipitated by A1 in Western blot (Fig. 6b). Again, A1 did not bind this protein in Western blots, indicating that A1 may bind to Kit via a conformational epitope. In keeping withthe observation that Kit is internalised following ligation by SCF [22], binding of A1 to HMC-1 (analysed by flow cytometry) was reduced when HMC-1 were exposed to SCF 100 ng/ml (Fig. 6c). Importantly, A1 bound to human Kit-expressing mouse MCBS1 cells [20] but not mock-transduced or untransduced E1-AA685 or wild type cells respectively (Fig. 7).

### scFV A1 does not interfere with Kit signalling

**Fig. 8:**
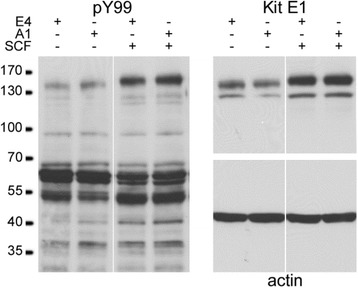
scFv A1 does not initiate or interfere with Kit signalling. HMC-1 were incubated with scFv A1 or E4 control in the presence or absence of SCF 100 ng/ml. Total intracellular phosphorylation assessed by Western blotting (PY99 antibody) was not altered by A1 in either the absence or presence of SCF. Phosphorylation of Kit (bands 145/120 kDa) in the left panel was also not altered. Staining for Kit (E1, right panel) and β-actin demonstrates equal loading for Kit within each pair of experiments. Representative of three independent experiments

Incubation of HMC-1 with A1 (20 μg/ml) did not induce the phosphorylation of Kit or other intracellular proteins, and did not inhibit SCF-dependent phosphorylation of Kit or other intracellular proteins when compared to the control scFv E4 (Fig. 8). This suggests that A1 most likely binds to Kit outside the SCF-binding domain. However, epitope mapping will be required to confirm this.

## Discussion

By immunising rabbits with membrane proteins derived from HLMC and HMC-1 cells, we were able to identify scFv-expressing phage which bound to HMC-1 but not Jurkat cells. Subsequent purification of several HMC-1-specific scFv antibodies identified an antibody, A1, that binds to Kit, and which is able to attenuate HMC-1 and HLMC adhesion to both HASMCs and BEAS-2B airway epithelial cells.

Cell-cell adhesion is a fundamental mechanism through which cells communicate. HLMC and HMC-1 adhere to human airway epithelial cells, HASMCs and lung fibroblasts [5, 6, 8, 23], and human skin MCs and HMC-1 cells adhere to skin fibroblasts [7]. In these previous studies, extensive investigation was undertaken to identify the adhesive mechanisms, but virtually all adhesion pathways known at the time were not operative. However, we could demonstrate that O-glycosylation was involved in the adhesion of human MCs to airway epithelium [6]. Subsequently we showed that HLMCs and HMC-1 cells adhere to HASMCs, and that this occurs in part via CADM1 [5]. Therefore much is still not known about the adhesive mechanisms mediating the above interactions. In all of these studies, the immature leukaemic HMC-1 cell line shared similar adhesion properties to the differentiated cells from lung and skin. We therefore generated scFv antibodies to human MC membrane proteins using the technique of phage display in an attempt to identify further adhesion pathways in HLMCs and HMC-1.

In mice, MC adhesion to fibroblasts is mediated in part via MC-expressed c-kit binding to membrane-bound SCF on fibroblasts [24], and in part by CADM1 expressed by MCs acting heterophilically [25]. Human gut MCs adhere to human umbilical vein endothelial cells (HUVEC) via a Kit/SCF-dependent mechanism [26], which is attenuated by function-blocking antibodies to either Kit or SCF, indicating an adhesive interaction between Kit and membrane-bound SCF. However, similar function-blocking antibodies did not demonstrate a role for Kit/SCF in the adhesion of HLMCs or HMC-1 cells to HASMCs or airway epithelium [5, 6, 8], or human skin MCs or HMC-1cells to skin fibroblasts [7]. Interestingly, our scFv antibody A1 detects Kit, and partially attenuates HMC-1 and HLMC adhesion to both BEAS-2B human airway epithelial cells and HASMCs, suggesting that Kit-dependent adhesion does in fact contribute to HMC-1 adhesion to these cells. Because A1 does not interfere with SCF-dependent Kit signalling, and because several studies have shown that function-blocking SCF/Kit antibodies do not inhibit HMC-1, HLMC or skin MC adhesion, it is likely that the A1 antibody binds outside the SCF binding domain on Kit. If true, then this indicates the presence of a novel adhesion epitope on human Kit, which mediates adhesion via a counter-receptor which is not SCF.

Such a mechanism is highly plausible because Kit is a type III receptor tyrosine kinase which is a member of the immunoglobulin (Ig) superfamily, with 5 extracellular Ig-like domains. The SCF binding site is situated in the three N terminal Ig domains, with the core in the second domain [27]. Kit is potentially glycosylated at N and O glycosylation sites although the nature of Kit glycosylation is poorly defined. Both N and O glycans are involved in adhesion mediated by many receptors. Our A1 antibody was only able to detect native Kit as shown by immunoprecipitation and flow cytometry, and not under reducing conditions used for Western blotting. This suggests that this antibody recognises a distinct conformational epitope.

## Conclusions

In summary, we have generated a Kit-specific antibody which partially attenuates human MC adhesion to both human airway epithelial cells and HASMCs. Our results indicate that Kit may contribute to these adhesion pathways via a previously unidentified adhesion epitope. Targeting this adhesion pathway might offer a novel approach for the inhibition of MC interactions with structural airway cells, without detrimental effects on Kit signalling in other tissues.

## Abbreviations

ASM: Airway smooth muscle
HASMC: Human airway smooth muscle cell
HLMC: Human lung mast cell
MC: Mast cell
scFv: single chain Fv antibody
SCF: Stem cell factor
